# The first case of *Acrophialophora levis*-induced severe pneumonia: a case report and literature review

**DOI:** 10.1186/s12879-019-4528-8

**Published:** 2019-10-15

**Authors:** Junyun Huang, Zhiping Liu

**Affiliations:** 1 Department of Laboratory Medicine, The First-affiliated Hospital of Gannan Medical University, Ganzhou, 341000 Jiangxi China; 2 School of Basic Medicine, Gannan Medical University, Ganzhou, 341000 Jiangxi China

**Keywords:** *Acrophialophora*, Human infection, Severe pneumonia

## Abstract

**Background:**

In recent years, some rare fungi have been increasingly recognized as new human pathogens. Here we reported the first fatal case of human severe pneumonia complicated by multiple organ dysfunction caused by *Acrophialophora levis* infection*.* However, its pathogenic mechanism and risk factors are unknown. *Acrophialophora* genus has only reported in six cases of human infection worldwide, but it has not been reported previously in China.

**Case presentation:**

A 71-year-old male patient with severe pneumonia complicated with multiple organ dysfunction caused by *A. levis* infection. The fungal identification was based on micromorphology and sequence analysis of the internal transcriptional spacer (ITS) of ribosomal RNA genes recovered from lower respiratory tract secretions. The microbial characteristics, sensitivity to antifungal drugs of this isolated *A. levis* were studied. Anti-infective regimen, liposomal amphotericin B combined with tegacycline, was used to prevent infection. The next day, the fever decreased, body temperature fluctuated between 36.5 and 37.8 degree, cough and sputum decreased, and sputum volume decreased, with oxygen uptake for 5 L/min, blood oxygen saturation over 95%. After 17 days of treatment, CT reexamination showed that the lesions in the right lung and left upper lung were absorbed and pleural effusion was reduced. The next 8 days, the patient asked to return to the local hospital for treatment. The local hospital stopped using liposomal amphotericin B because of the absence of liposomal amphotericin B, and died of respiratory failure 2 days later.

**Conclusions:**

This study is the first to report the occurrence, risk factors, molecular determinants, microbial characteristics and susceptibility to antifungal agents of *A. levis* infection in China. In addition, six published cases of human infection with *Acrophialophora* were reviewed.

## Background

In recent years, immunosuppressive drugs in organ transplantation, chemotherapy drugs in cancer, and corticosteroids have been widely used. Furthermore, the number of HIV patients, invasive tests in vivo, aging populations, patients with diabetes and other chronic diseases have been on the rise. Therefore, the morbidity and mortality of invasive fungal infection increase year by year, which become serious threatens to human health. Some rare fungi are increasingly recognized as new human pathogens.

*Acrophialophora* is a rare opportunistic and heat-resistant soil pathogenic fungus in temperate and tropical zones. Human infection with *Acrophialophora* has been rarely reported and its pathogenicity is largely unknown. This genus was originally classified as *Paecilomyces*, but these two fungi are obviously different, because the colonies of *paecilomyces* never form black colonies. However, they have the similar morphology under microscope and are difficult to distinguish. Until 2015, Marcelo Sandoval-Denis et al., made use of the sequence analysis of the large subunit (LSU) of ribosomal DNA, internal transcribed spacer (ITS) and fragments of β tubulin (Tub)gene, and then determined that *Acrophialophora* belongs to *Chaetomiaceae,* including three closely related species named *Acrophialophora fusispora*, *Acrophialophora levis* and *Acrophialophora seudatic* [1]. These three species lack characteristic differences. The conidia of *A. fusispora* are brown oval to spindle-shaped and have a three-dimensional rough spiral stripe pattern. The conidia of *A. levis* are transparent oval to cylindrical and have smooth and fine spiral stripe pattern. However, *A. fusispora* and *A levis* are only slightly different in conidia, which cannot support the conclusion of genus identification. Using molecular diagnostic method, Marcelo et al., found that the LSU sequences (99.9%) of the three strains in *Acrophialophora* were very similar, but the sequences of ITS and Tub were very different respectively about 96.1 and 96.6%), indicating that ITS and Tub were more conducive for the identification of species [1].

## Case presentation

**Fig. 1:**
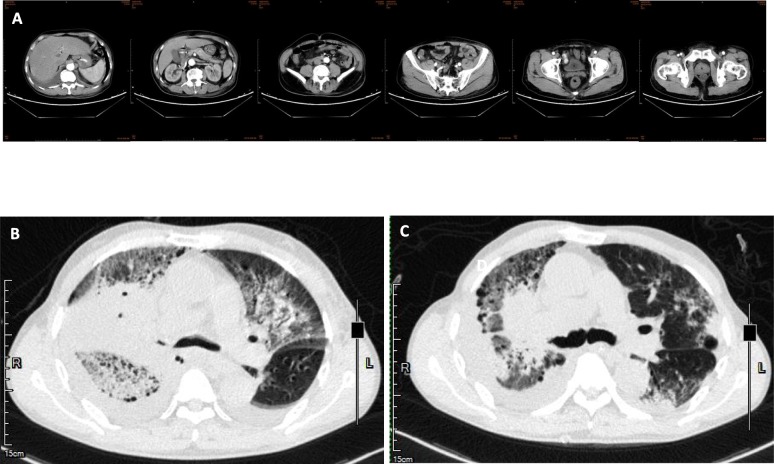
Chest CT results of *Acrophialophora* infection. **a** no abnormal images were found in the enhanced CT scan of the whole abdomen on September 5, 2018. **b** Chest CT images prior to treatment. **c** Chest CT images after treatment with liposomal amphotericin B

A male, 71 years old, a retired teacher, did not have previous history of diseases, including hypertension, diabetes, coronary heart disease, chronic lung disease, kidney disease, and liver disease. He had a long history of smoking with 20 cigarettes per day. On August 24 2018, he got a fever with the heat peak at 40.0 °C without known causes and heat type, accompanied by chills, dizziness, abdominal pain, cough, and by a lot of yellow purulent sputum which was occasionally bloody. These symptoms appeared mainly in the morning and night, accompanied by right chest pain, aggravating when coughing, and difficulty in breathing. After an ineffective antibiotic treatment in the local hospital, he was then transferred to our hospital on September 2nd, 2018. The Computed Tomography (CT) results showed a large consolidation, grinding glass shadow, honeycomb changes, lung balloon formation in the right lung, and a newly-discovered solid patch and grinding glass shadow in the left, as well as bilateral pleural effusion (Fig. 1). These indicate: 1. Double lung infection, interstitial pneumonia (mainly right lung), left emphysema, pneumatocele in the upper lobe of left lung; 2. Bilateral pleural effusion, mainly in the right lung; Laboratory analysis and display: Blood analysis: white blood cells 17.34 × 10^9^ / L, neutrophil count 15.52 × 10^9^ / L, neutrophil ratio 89.5%, lymphocyte count 0.83 × 10^9^ / L, lymphocyte ratio 4.8%, platelets 102 × 10^9^ / L. Abnormal test results in liver function: Alanine aminotransferase (ALT) 709 U/L, Aspartate aminotransferase (AST) 474 U/L, Cholinesterase (CHE) 2789 U/L, Total bilirubin (TB) 25.6umol/L, Direct Bilirubin (DB) 23.3umol/L, Lactate dehydrogenase (LDH) 758 U / L; Abnormal test results in renal function: Blood urine nitrogen (BUN) 10.10 mmol / L, Creatinine (Cr) 122umol / L; Hypersensitivity C reactive protein (Hs-CRP) 17.49 mg / dl. Abnormal test results in coagulation function: Prothrombin time (PT) 17.40, international normalized ratio (PT-INR) 1.52, D-dimer (DD) 2.37 mg/L; sputum anti-acid staining (−), T-SPOT (−), HIV antibody (HIV-Ab) (−); The patient was diagnosed to be severe pneumonia with multiple organ dysfunction. According to the initial experience, caspofungin (CAS) combined with imipenem, moxifloxacin and oseltamivir were given to resist infection; At 7 days later, the re-examination of chest CT plain scan showed that the pulmonary lesion did not change much in comparison with the previous symptom, showing that the treatment was ineffective, and the clinical symptoms did not improve.

**Fig. 2:**
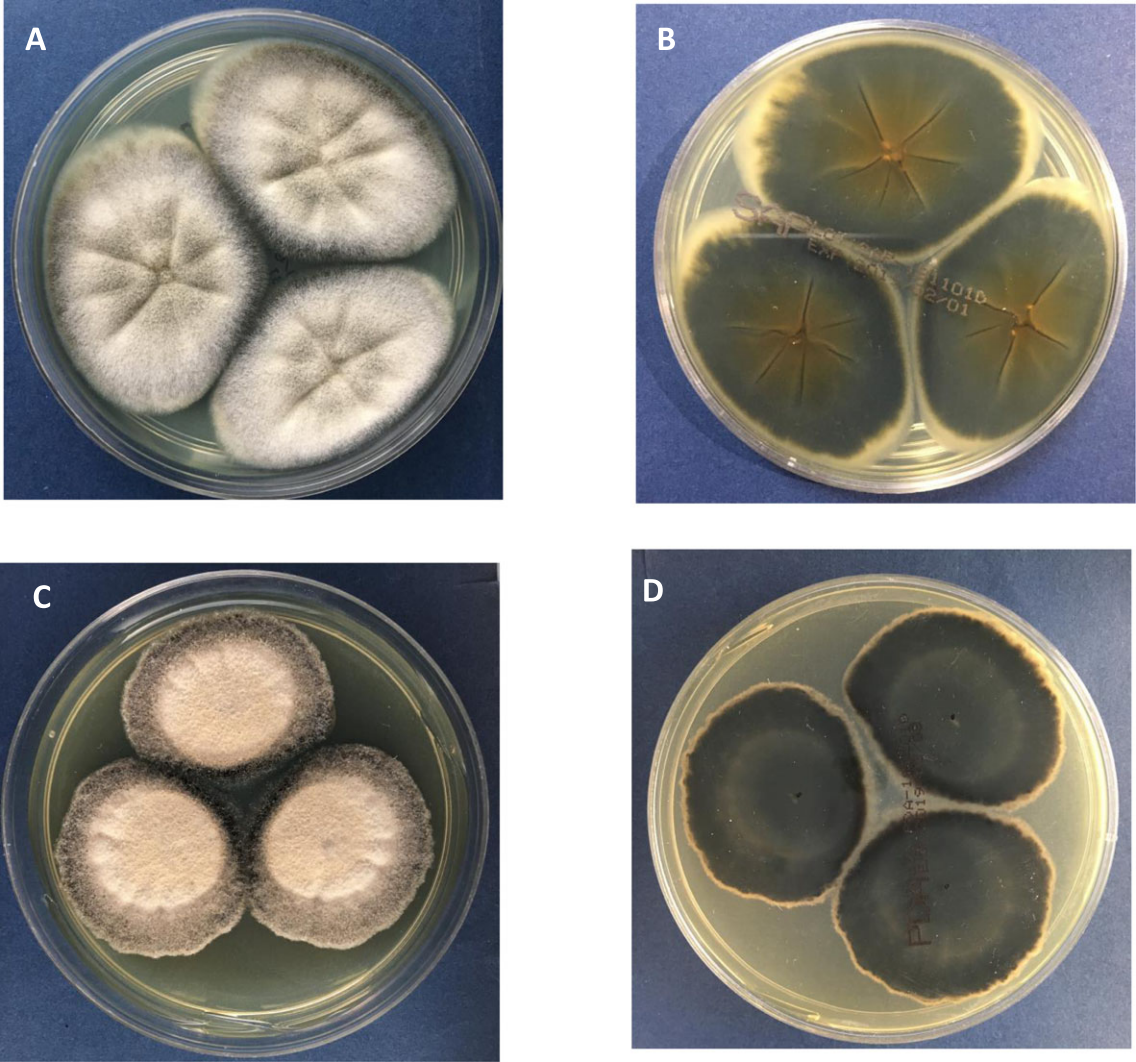
Colony morphology of *A. levis* (**a**) SDA-28 degrees -- 6 days (colony front). **b** SDA-28 degrees −6 days (on the back of the colony). **c** PDA-28 degrees − 6 days (colony front). **d** PDA-28 degrees − 6 days (back of colony)

**Fig. 3:**
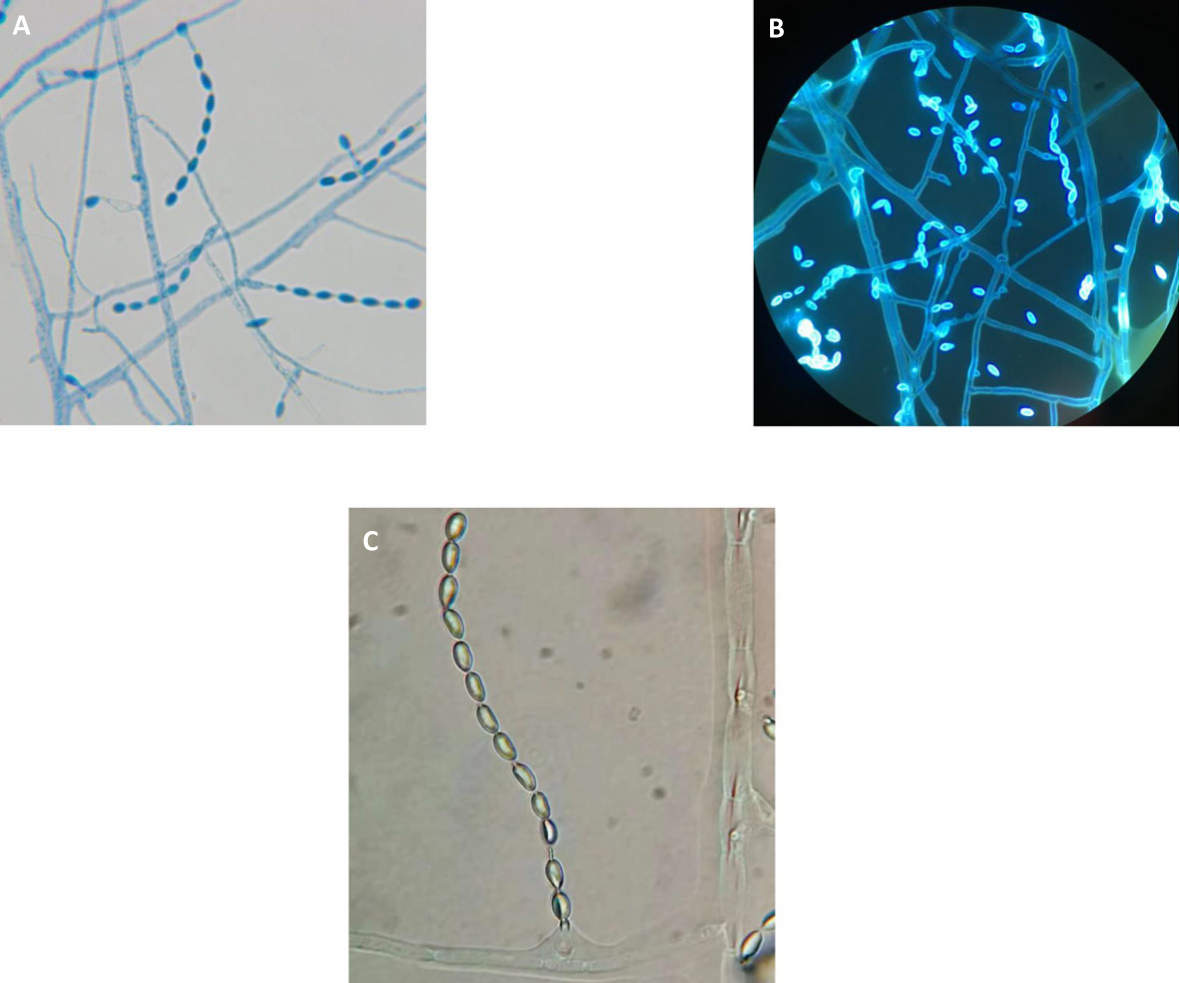
Microscopic morphology of *A. levis* (**a**) lactate phenol-cotton orchid staining (× 1000). **b** fluorescence staining (× 1000). **c** small culture for 3 days (× 1000)

Mycelia and spores were found frequently in the sputum samples of the lower respiratory tract by Gram staining and methylenol lactate microscopic examination. Colonies could be observed in fungal culture: the Sabourauds Agar (SDA) and Potato Dextrose Agar (PDA) showed rapid growth at 25 °C, 35 °C and 42 °C, especially on the SDA, with colonies of a gray front and a black reversed. It was dark grayish brown on the PDA. The characteristics of colonies on various substrate were shown in Fig. 2. Microscope: the hyphae was light brown, 1.5–3.5 μm wide, straight, separated, and unbranched. Erected or slightly curved conidiophores stretch out from the top or side of the hyphae. The tip was tapered; the base was slightly enlarged with a bottle-shaped stalk; the long and narrow neck was similar to the tubular of the *Paecilomyces*, appearing transparent, and smooth. An array of oval or round conidium was produced from the top of or directly from the end or side of conidiophores arranged in chain. Elliptical conidial was a unicellular microorgnism, which was straight or slightly curved, tapered to the top, transparent, smooth, 4–9 μm long, 2–6 wide micron with a smooth fine spiral strip pattern. The various staining and morphological features under microscopy were shown in Fig. 3.

For further identification, a single colony on the SDA purification plate was selected, and the general primers for ITS1 and ITS4 of fungi were used. By amplying the region of the internal transcriptional interval region, the sequencing results were analyzed by BLAST comparison. The homology to the *Acrophialophora levis* sequence in the gene bank was 99%, and the NCBI number referenced was KM995879. We have also submitted the sequence data to GenBank (Accession number is MN461541).

After that, sensitivity to antifungal drugs was further tested by colorimetric microdilution method recommended by CLSI M61 version 2018. The tested antifungal drugs were amphotericin B (AMB), 5-flucytosine (5-FC), anidulafungin (AND), CAS, micafungin (MF), fluconazole (FLU), itraconazole (ITR), posaconazole (PSC), and voriconazole (VRC). Until now, the in vitro sensitivity information of *Acrophialophora* had not been reported. Among them, AMB (≤0.12 μg/ml) had the best anti-fungal activity against *A. levis* in vitro, following by the activity of VRC (0.12 μg/ml), ITR (0.12 μg/ml), and PSC (0.25 μg/ml) in vitro. The activity of 5-FC (≥64 μg/ml), AND (≥8 μg/ml), CAS (≥8 μg/ml), MF (≥8 μg/ml), and FLU (16 μg/ml) were poor. Drug sensitivity results confirmed that the original drug, CAS, was not effective. Then liposomal amphotericin B was used for suppressing infection. After that, the fever of patient declined with fluctuations from 36.5 to 37.8 °C. He had reduced sputum volume, cough, sputum blood in phlegm, but still had the white phlegm, with oxygen 5 L/min and blood oxygen saturation by more than 95%. The re-examination of chest CT plain scan at 17 days after the change of anti-infection program indicated that the upper left pneumonia sites were more reduced than before, and the pleural effusion was less severe than before. The next day, the patient was asked to go back to the local hospital for treatment. The treatment was discontinued because liposomal amphotericin B was not available in the local hospital, and this patient died of respiratory failure at 2 days later. The imaging data were shown in Fig. 1.

## Discussion and conclusions

*Acrophialophora* is a heat-resistant, fast-growing opportunistic fungus, which is widely distributed in temperate and tropical zones. Its pathogenesis and risk factors are unknown. Shukla PK et al. reported for the first time in 1983 the experimental corneal lesions in animals caused by *A. fusispora* infection, which confirmed that *A. fusispora* was pathogenic [2]. *Acrophialophora* is now becoming a known pathogen of human and animals [3].

### Literature review

Except 4 reported cases of airway colonization of *Acrophialophora*, only 6 cases of human *Acrophialophora* infection had been reported (Table 1). There were no cases of *Acrophialophora* infection in China Mainland, and the *A. levis* infection in this paper is the first report from China. From Table 1, *Acrophialophora* mainly caused keratitis in 2 cases [3, 6], pulmonary infection in 2 cases [3], and brain infection in 2 cases [4, 5]. Among the two reported cases of human keratitis, one was caused by corneal scratches caused by sawdust in Indian woman and the other was a Japanese male patient, possibly due to pulmonary keratitis. One of the two cases of brain abscess was a child with acute lymphoblastic leukemia, who suffered from cavitary lung lesions and brain abscess after chemotherapy. *A. fusispora* was a neurotropic fungus in this case report. The second case was a patient with HIV infection who had brain abscess secondary to cryptogenic tissue pneumonia. To sum up, among 6 reported cases of *Acrophialophora* infection plus this case, a total of 6 cases were originated from the lungs. Again, it was confirmed that the common organs involved in *Acrophialophora* infection were the lungs. It was speculated that the eyes and airways might be the main route of this fungal infection, which was consistent with the previous reports. Five of the 7 infected patients had severe immune hypofunction. Immune defect is a risk factor for infection. The reported *A. levis*-infected patient in this case report was a retired teacher with a long history of smoking with 20 cigarettes a day. Before the onset of the disease, the old house was demolished and ready to be rebuilt. His infection was possibly originated from the dust flying from the demolished old house. The infection mode of *A. levis* soil fungi was in line with *Acrophialophora* infection mode by inhaling the soil fungi *A. levis*.

**Table 1:** The comparison of reported human cases of *Acrophialophora* infection

Case	Time	Countries	Gender/Age	Basic diseases	Diseases	Fungal type	Pathogen identification	Pathogen sites	Antifungal drug sensitivity	Outcome	References
1	1997	Spain	Male/67	Pulmonary fibrosis bullous emphysema; lung transplantation	Lung infection	*A.fusispora*	Morphological identification	Lower respiratory tract secretions	AMB Liposome, ITR	Dead	Sutton et al.
2	1998	Sudan	Female/12	Acute lymphocyte leukemia	Brain abscess	*A.fusispora*	Morphological identification	Cerebrospinal fluid aspirate	AMB Liposome, ITR	Survive	Al-Mohsen et al. [4]
3	2002	Portugal	Male/33	lung transplantation	Lung infection	*A.fusispora*	Morphological identification	Sputum, bronchoalveolar lavage fluid	VRC	Improved	Guarro et al. [4]
4	2005	India	Female/55	None	Fungal keratitis	*A.fusispora*	Morphological identification	Corneal scrape	FLU	Therapeutic keratoplasty rehabilitation after ineffectiveness	Guarro et al. [3]
5	2010	Taiwan	Male/60	Acquired immunodeficiency syndrom	Brain abscess	*A.fusispora*	Molecular diagnosis	Cerebrospinal fluid aspirate	VRC	Dead	Chia-Wen Li et al. [5]
6	2018	Japan	Male/77	Neutropenia and Prostatic Cancer	Corneal ulcer and uveitis infection	*A.spp*	Molecular diagnosis	Corneal scrape	AMB Liposome、ITR is not effective, then VRC was used	Cured	Watanabe Y et al. [4]
7	The current Report	China	Male/71	None	Severe pneumonia	*A.levis*	Molecular diagnosis	Lower respiratory tract secretions	AMB Liposome、Caspofungin	Dead	

Of the 6 reported human infections in Table 1, only 2 cases were confirmed by sequencing in ITS region, and the other 4 cases were confirmed only by morphology, lacking of molecular diagnostic evidence, which may lead to a false positive detection. The detected *A. levis* was by ITS sequencing verification, and the results are true and reliable.

In addition, 4 cases of temporary or chronic airway colonization of *A. fusispora* in patients with cystic fibrosis (CF) were reported [7]. Because the clinical condition of the patients did not change significantly and there was no evidence of infection, it was not included in current paper. However, airway colonization of *A. fusispora*, especially in long-term colonization cases, can also lead to progressive lung injury. The prevalence of *Acrophialophora* in CF patients should not be underestimated.

Because of the rarity of *Acrophialophora*, the data of antifungal sensitivity tests are very few, and the available clinical outcomes are very limited. The clinical treatment reference is mainly based on previous case reports. Therefore, the clinical & laboratory standards institute (CLSI) is currently unable to give a clinical breaking point which has a strong indication for clinical outcomes. The in vitro sensitivity of isolates to antifungal drugs can only be estimated by comparing the concentration and dosage of antifungal drug minimum inhibitory concentrations (MICs) or minimum lethal concentrations (MLCs) in the serum of patients [8, 9]. *A. levis* is the first to be detected in this case report and has no comparable historical results. It can only be compared to the antifungal sensitivity data of 4 cases of human infection with *Acrophialophora* by literature meta-analysis (see Table 2). The results showed that four *Acrophialophora* isolates were highly sensitive to VRC, while all other drugs showed poor activity against these fungi in vitro. VRC could be considered for the treatment of these serious infections. And 2 cases were cured by VRC [3, 6]. The antifungal sensitivity of *A. levis* in this case is different from previous results. AMB has the best anti-fungal activity in vitro, the worst activity to echinococcin and fluorouracil, the good activity to three azole ITR, but the poor activity to FLU, and the second generation of triazole, VRC and PSC (Table 2). CAS was ineffective in the treatment of this patient. After 17 days of liposomal amphotericin B treatment, lung infections have improved markedly, which was consistent with the above experimental conclusions. Watanabe Y et al. [7] pointed out that invasive *Acrophialophora* infection may require long-term treatment. The patient died of respiratory failure after discontinuation of liposomal amphotericin B due to over optimistic estimation. The current data are not sufficient and further data are needed to confirm these results in the future.

**Table 2:** The sensitivity test results of an-fungal drugs to *Acrophialophor*a ^a^

Case#.	Fungi	MIC ^b^ (μg/ml) of drug at 72 h^c^								
		AMB	5-FC	FLU	ITR	VRC	MF	PSC	AND	CAS
Case 1	*A.fusispora*	4	NT	NT	NT	0.25	>128	0.25	NT	NT
Case 2	*A.fusispora*	1.0	>64	NT	0.25	NT	NT	NT	NT	NT
Case 4	*A.fusispor*a	2	NT	NT	NT	0.25	>128	0.25	NT	NT
Case 6	*A.spp*	5.66	NT	NT	NT	0.17	NT	NT	NT	NT
Case 7	*A.levis*	≤0.12	≥64	16	0.12	0.12	≥8	0.25	≥8	≥8

*NT* Not tested

^a^Tested in Thermo Fisher Yeast One plate, 35 °C

^b^MICs are defined on the basis of the first tube showing no growth (AMB) or the first tube with an 80% reduction in growth (the azoles and 5F-C) compared to the drug-free control

^c^Tubes were read after 72 h of incubation

## Data Availability

All data generated or analyzed during this study are included in the published article.

## Abbreviations

5-FC: 5-flucytosine
ALT: Alanine aminotransferase
AMB: Amphotericin B
AND: Anidulafungin
AST: Aspartate aminotransferase
BUN: Blood urine nitrogen
CAS: Caspofungin
CF: Cystic fibrosis
CHE: Cholinesterase
CLSI: Clinical & laboratory standards institute
Cr: Creatinine
CT: Computed Tomography
DB: Direct Bilirubin
DD: D-dimer
FLU: Fluconazole
Hs-CRP: Hypersensitivity C reactive protein
INR: International normalized ratio
ITR: Itraconazole
ITS: Internal transcribed spacer
LDH: Lactate dehydrogenase
LSU: Large subunit
MF: Micafungin
MICs: Minimum inhibitory concentrations
MLCs: Minimum lethal concentrations
PDA: Potato Dextrose Agar
PSC: Posaconazole
PT: Prothrombin time
SDA: Sabourauds Agar
TB: Total bilirubin
TUB: Tubulin
VRC: Voriconazole
